# Nucleosomes determine their own patch size in base excision repair

**DOI:** 10.1038/srep27122

**Published:** 2016-06-06

**Authors:** Rithy Meas, Michael J. Smerdon

**Affiliations:** 1 School of Molecular Biosciences, Washington State University, Pullman, WA, USA.

## Abstract

Base excision repair (BER) processes non-helix distorting lesions (e.g., uracils and gaps) and is composed of two subpathways that differ in the number of nucleotides (nts) incorporated during the DNA synthesis step: short patch (SP) repair incorporates 1 nt and long patch (LP) repair incorporates 2–12 nts. This choice for either LP or SP repair has not been analyzed in the context of nucleosomes. Initial studies with uracil located in nucleosome core DNA showed a distinct DNA polymerase extension profile in cell-free extracts that specifically limits extension to 1 nt, suggesting a preference for SP BER. Therefore, we developed an assay to differentiate long and short repair patches in ‘designed’ nucleosomes containing a single-nucleotide gap at specific locations relative to the dyad center. Using cell-free extracts or purified enzymes, we found that DNA lesions in the nucleosome core are preferentially repaired by DNA polymerase β and there is a significant reduction in BER polymerase extension beyond 1 nt, creating a striking bias for incorporation of short patches into nucleosomal DNA. These results show that nucleosomes control the patch size used by BER.

Human cells encounter endogenous and exogenous stresses that create a wide range of DNA lesions. A major subset of these are non-helix distorting DNA lesions (e.g., the misincorporation of uracil, alkylation of bases, and depurination of bases) that are repaired by base excision repair (BER)^1^. Ineffective removal of these lesions can lead to the accumulation of mutations and has been associated with a number of human disorders^2^.

In BER, removal of a chemically modified base is initiated by recognition of a DNA glycosylase that subsequently cleaves the N-glycosidic bond to release the base from the deoxyribose phosphate backbone. The resulting abasic site is recognized by an AP endonuclease (APE) that cleaves the DNA backbone 5′ of the abasic site leaving a 3′ hydroxyl and a 5′ deoxyribose phosphate. This nicked substrate can be processed by one of two BER subpathways: short patch (SP) or long patch (LP) repair. Processing via SP repair requires the DNA deoxyribophosphodiesterase (dRPase) activity of polymerase β (Pol β) to remove the 5′ deoxyribose phosphate moiety, resulting in a 1-nucleotide (nt) gap and a ligatable 5′ phosphate^3^,^4^. Pol β then adds 1 nt to the 3′ hydroxyl group that is subsequently ligated to the adjoining DNA strand^5^. In LP repair, Pol β, δ, and/or ε polymerize more than 1 nt and displace the 5′ deoxyribose phosphate strand^6^,^7^,^8^. This displacement creates a “flap” of DNA that is cleaved by flap endonuclease, which creates a 5′ phosphate strand that can be ligated to the 3′ hydroxyl extended strand^9^. Alternatively, Pol β and flap endonuclease can alternate cleavage and synthesis to create a 2- to 11-nt repair patch via a “Hit and Run” mechanism^10^. The choice to proceed via LP or SP BER of naked DNA has been shown to be influenced by the type of DNA glycosylase^11^ and ATP concentration^12^. However, the choice between these two BER subpathways has not been analyzed in the context of nucleosomes.

The canonical nucleosome core particle (NCP) contains ~147 base pairs (bps) of DNA wrapped 1.67 times around a histone octamer comprised of two H2A/H2B dimers and an H3/H4 tetramer. The NCP is an occlusive substrate for DNA repair because it restricts accessibility of repair proteins and has been shown to impact some BER steps: lesion recognition and ligation. The translational position (i.e., distance from the nucleosome dyad) and rotational position (i.e., proximity of the phosphate backbone to the histone octamer) of a lesion within an NCP or nucleosome (NCP plus linker DNA) affect accessibility by DNA glycosylases and APEs^13^,^14^,^15^,^16^,^17^. For example, uracils located close to the nucleosome dyad or rotated toward the core histones are processed less efficiently than uracils close to the entry/exit site of the nucleosome or rotated toward the solvent^14^,^15^,^16^. While flap endonuclease activity is not adversely affected in nucleosomes as compared to naked DNA^18^, in the final step of LP BER, DNA ligation by DNA ligase I is inhibited by occlusive translational positions along the nucleosome^19^. In contrast, the mechanism(s) of the BER synthesis step in nucleosomes is not well understood; therefore, this report addresses how nucleosome structure impacts DNA polymerase extension and BER patch size.

In the present study, we analyzed how the polymerase extension step of BER is affected by the translational and rotational positions of repair sites in NCPs and nucleosome linker DNA. We find that only 1 nt is inserted during BER when uracil (or a 1-nt gap) is positioned within the NCP, whereas polymerase extension readily proceeds past 1 nt when a uracil or a gap is situated in linker DNA. Importantly, this bias for 1-nt extension by BER occurs only up to the NCP boundary. These results indicate that DNA polymerases other than Pol β have markedly reduced accessibility to repair sites within NCPs. This marked bias may reflect different structural constraints of the template strand for each of these polymerases during repair patch extension in nucleosomes.

## Results

### ‘Designing’ nucleosomes with DNA lesions at different rotational and translational settings

**Figure 1:**
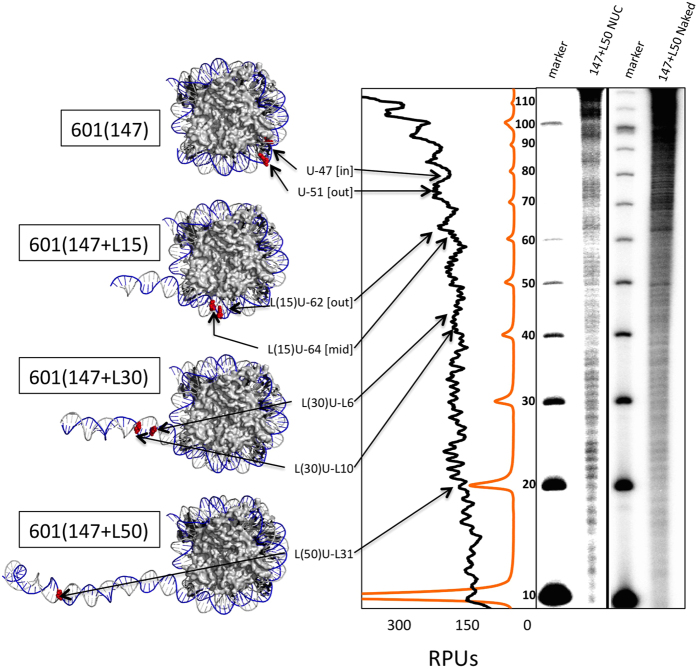
Uracil position along the 601 NCP and nucleosome (NUC). The 147 bp 601 NCP crystal structures (PDB: 1ZBB) with different lengths of DNA linker (0 bp, 15 bp, 30 bp, and 50 bp) are shown, and the names indicate the composition of the nucleosome. For example, 601(147 + L30) denotes 147 bps of the 601 positioning sequence with addition of 30 bps of linker DNA. The nomenclature used in the subsequent figures indicates the position of the uracil (red base) relative to the DNA dyad or end of the NCP DNA (e.g., U-47 is 47 bp from the dyad and L(30) U-L10 is 10 bp into the 30 bp linker DNA from the nucleosome core). The rotational positions of uracil in the NCP are indicated in brackets ([in], [mid], or [out]). The •OH radical footprint (right side of figure) of 601(147 + L50) nucleosome and naked DNA is plotted as relative phosphorimager units (RPUs) for the nucleosome DNA (black line) and 10 bp DNA marker (marker scaled 1:10; orange line). Arrows mark the corresponding positions of uracil sites on the crystal structure and along the relative intensity graph.

The accessibility of DNA in nucleosomes to DNA glycosylase and APE is not uniform. Rather, accessibility depends on both the translational and rotational position of the lesion within the nucleosome^15^. Therefore, we positioned a lesion (either uracil or a 1-nt gap, denoted as “U” or “G,” respectively) at different translational and rotational positions within the NCP region of the Widom 601 positioning sequence^20^ or within adjacent linker DNA not associated with the nucleosome core (Fig. 1 and Supplementary Fig. S1). Bases within this sequence are labeled relative to the dyad center of the NCP, termed “0”. For example, “U-47 NCP” denotes a NCP DNA substrate that contains a uracil located 47 nts from the dyad and “L(30)G-L6 NUC” denotes a nucleosome substrate containing a 30-bp linker with a gap 6 nts from the edge of the 147 bp NCP. The rotational positions of the lesions were verified by •OH footprint analysis, a DNA-reactive process that reveals locations of associated protein that prevent •OH-induced cleavage, in the DNA of the longest substrate (147 + L50, containing the 147 bp 601 sequence and a 50 bp linker DNA). Our results show the typical ~10 bp periodicity found in nucleosomes^15^,^21^, where the solvent exposed phosphate backbone [out] is readily cleaved as compared to regions that are partially solvent exposed [mid] or facing toward the histone octamer [in]. Interestingly, the ~10 bp periodicity appears to extend from the NCP to ~30 bp of the linker DNA [Fig. 1; and ref. 21], though the footprint is not as pronounced in the linker as compared to NCP DNA. This periodicity is not seen in the naked DNA substrate, indicating far-reaching interactions between the histone octamer and linker DNA.

### Restricted BER cleavage in the nucleosome core

**Figure 2:**
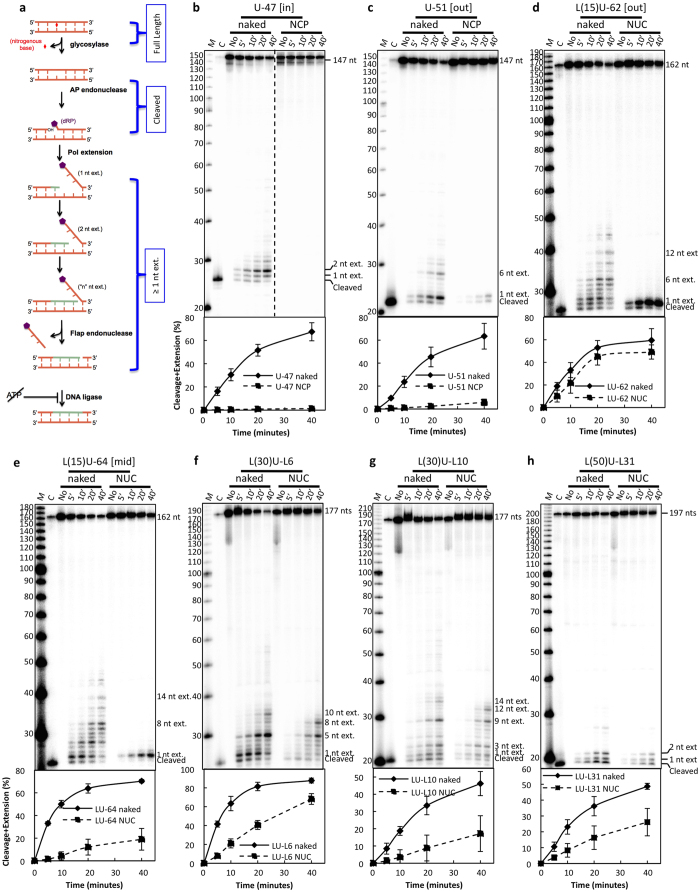
Uracils located in nucleosome core DNA affect BER cleavage and polymerase extension. (**a**) Schematic of the BER cleavage and polymerase extension assay: BTNE and dNTPs are incubated with DNA substrates in the absence of ATP and expected DNA products are shown. Samples were run on denaturing sequencing gels to compare naked DNA, NCP, and NUC of different uracil positioning sequences: (**b**) U-47, (**c**) U-51, (**d**) L(15) U-62, (**e**) L(15) U-64, (**f**) L(30) U-L6, (**g**) L(30) U-L10, and (**h**) L(50) U-31. The 10 bp size marker “M”, UDG/APE1 cleaved control “C”, and the DNA products are labeled adjacent to the phosphorimager scan. Overall cleavage [Cleavage + Extension (%)] is the sum of the cleavage and extension product band intensities divided by the total intensity of the lane. Standard deviations of 4 replicates from 4 independent nucleosome reconstitutions are shown.

Previous studies have shown that BER is fully functional in various mammalian nuclear extracts^22^,^23^. In our assays, naked and nucleosome DNA substrates containing a U:A bp at various nucleosome positions were incubated with bovine testis nuclear extracts (BTNEs). Since the only ATP-dependent BER enzyme is DNA ligase^12^, we can specifically analyze polymerase activity by omitting ATP (Fig. 2a) and resolving the DNA products on DNA sequencing gels. The omission of ATP prevents DNA ligase activity; therefore, DNA ligation will not restrict polymerase extension; instead, the lack of ATP will promote strand displacement. Since the extended products must be cleaved before they are extended, total cleavage can be calculated from the sum of both the cleaved and extended products. In agreement with previous studies^14^,^15^,^16^, we find that when the lesion is in the NCP, DNA cleavage is significantly inhibited in (a) nucleosome substrates compared to naked DNA, (b) nucleosome substrates where the translational position of the uracil is closer to the DNA dyad, and (c) nucleosome substrates where the rotational position of the uracil is toward the histone octamer (Fig. 2b–e).

### BER polymerase extension is limited in nucleosome cores

Previous studies of BER polymerase extension in the context of nucleosomes used a single dNTP for incorporation, thus providing an “all or nothing” effect (i.e., if there is polymerase extension or not)^13^,^15^,^24^,^25^. A variable extension patch size at the AP endonuclease-cleaved sites has not been previously considered for nucleosomes. Interestingly, when all four dNTPs are available for BER synthesis and uracil is located in NCP DNA (i.e., not linker DNA), polymerase extension after cleavage by uracil DNA glycosylase and APE is limited to only one nucleotide (Fig. 2c–e).

### Restricted BER cleavage and polymerase extension in nucleosome linker DNA

The chromatinized genome also contains “linker” DNA between NCPs; however, BER in linker DNA is not well characterized. Therefore, we investigated BER-associated DNA cleavage and extension in linker DNA as well as DNA extension from the linker into the NCP. To measure cleavage and extension in linker DNA, we created a U:A bp at different positions within linker DNA (Fig. 1) and treated these naked and nucleosome-containing DNA substrates with BTNE in the absence of ATP (Fig. 2f–h) using the same assay with substrates containing uracils in the NCP (Fig. 2b–e). When uracil is located in linker DNA, cleavage decreased in nucleosome substrates as compared to the corresponding naked DNA (Fig. 2f–h). Importantly, while only 1-nt extensions are seen at lesions in the NCP (Fig. 2c–e), multi-nucleotide extension is observed in nucleosome substrates when uracil is located in the linker (Fig. 2f–h).

To analyze how far BER polymerase synthesis extends into NCPs, we first analyzed the polymerase extension profiles for L(30)U-L6 naked and nucleosome substrates (Fig. 2f). Interestingly, polymerase extension is greatly inhibited when extending past 8 nts in L(30)U-L6 NUC, which indicates the BER DNA polymerase(s) synthesize 6 nts of linker DNA, then stall once they have extended 2 nts into the NCP. Indeed, repositioning the uracil 4 nts further into linker DNA [L(30)U-L10 substrate] creates a 4-nt shift in the polymerase extension profile (compare Fig. 2f,g). Therefore, polymerase extension at the L(30)U-L10 NUC site is also strongly inhibited 2 nts into the NCP (Fig. 2g), suggesting there is a 2-nt limit for polymerase extension into the NCPs in our nucleosome substrates. Moreover, when the lesion is positioned further from the NCP in linker DNA (Fig. 2h), we do not observe stalling of BER polymerase extension, and the extension profiles between naked and nucleosome DNA are similar. Overall, these data indicate that both BER cleavage and polymerase extension in linker DNA are inhibited by the presence of an adjoining NCP and blocked from extending the repair patch beyond 2 nt into the NCP DNA.

### Nucleosome-dependent patch size in BER

**Figure 3:**
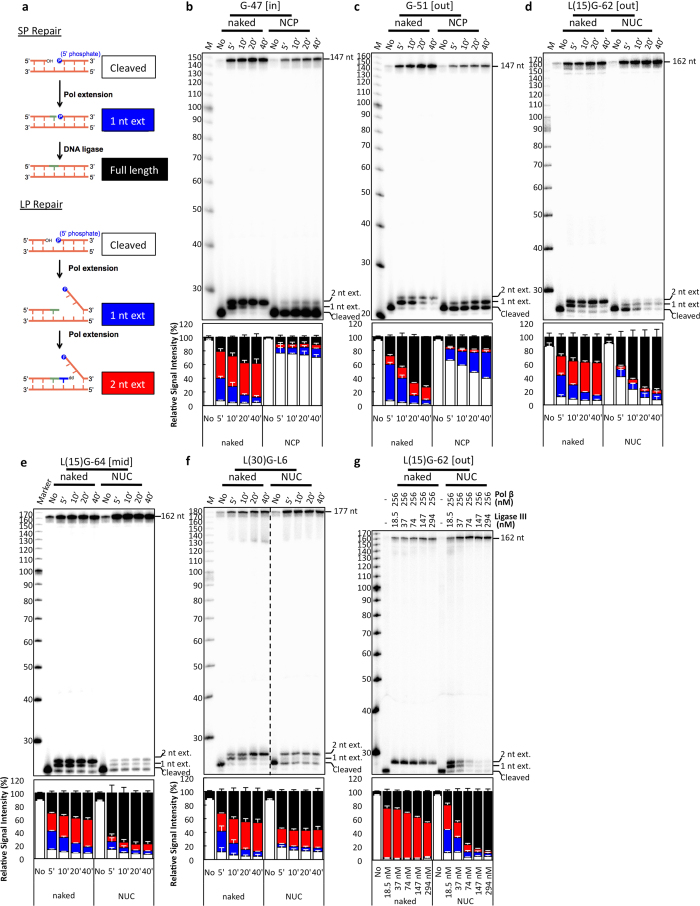
Gaps located in nucleosome core DNA are primarily repaired by SP BER. (**a**) Schematic of the SP/LP BER assay: 1-nt gap substrates were incubated in BTNE supplemented with ATP, dTTP, and a ddNTP, yielding four distinct DNA products to differentiate SP from LP repair. Samples from the SP/LP BER assay were run on denaturing sequencing gels to analyze (**b**) G-47, (**c**) G-51, (**d**) L(15) G-62, (**e**) L(15) G-64, and (**f**) L(30) G-L6 substrates. (**g**) SP/LP assay performed with purified Pol β, DNA ligase III, and the L(15) G-62 substrate for 20 minutes. Composite bar graphs (white, cleaved; red, 1 nt; blue, 2 nt; black, full-length) are plotted for each substrate. Quantification of the relative signal intensity of each of the four DNA bands is in Supplementary Tables S2–S7. Standard deviations of 3 replicates from 3 independent nucleosome reconstitutions are shown.

One of the most intriguing results seen in the BER polymerase cleavage and extension assay was the 1 nt extension limit that occurs in NCP DNA as compared to naked DNA (Fig. 2). Therefore, we hypothesized that repair of lesions located in the NCP is restricted to SP BER. To test this hypothesis, we devised an assay that distinguishes between SP and LP BER in BTNEs, which can carry out both of these BER activities^26^. Using a 1-nt gap substrate supplemented with dTTP, ddNTP, and ATP, the assay allows for SP repair to proceed fully after incorporating dTTP but stalls LP repair by incorporating ddNTP at the second position. This results in four different DNA products: 1) a cleaved, nonextended band indicating no DNA processing has occurred; 2) a 1-nt extended product indicating an intermediate for either SP or LP BER; 3) a full-length band indicating SP repair has occurred due to the incorporation of dTTP followed by DNA ligation; and 4) a 2-nt extended product indicating LP repair has occurred due to the incorporation of dTTP followed by a ddNTP (Fig. 3a).

In naked DNA, the substrates show no clear preference for SP or LP BER except G-51, which shows a bias toward SP repair (Fig. 3; Supplementary Tables S2–S7). When the gap is positioned closer to the dyad, conversion of the gap substrate to an extended product is reduced (Fig. 3b,c). However, when the gap is positioned closer to the nucleosome entry/exit site [L(15)G-62 and L(15)G-64 substrates], nearly all of the gapped substrate is converted to extended or ligated products after 40 minutes (Fig. 3d,e). Analysis of rotational orientation within similar translational positions indicate that G-47 [in] NCP obstructs incorporation of nucleotides more than G-51 [out] NCP (Fig. 3b,c).

There is a clear difference in the processing of gap substrates when they are closer to the dyad; however, analysis of the ratio between SP and LP repair of gaps that are located in the NCP indicate that SP repair is 2–15 times more prevalent than LP repair in NCP and NUC substrates (Fig. 3b–e). It was proposed that this preference may be facilitated by XRCC1, a DNA repair scaffold protein, which has been shown to disrupt nucleosomes^24^. However, when XRCC1 was immunodepleted (ID) from the BTNE, the preference for SP or LP BER was unaffected compared to mock ID BTNE (ID: control; Supplementary Fig. S2) indicating that the role of XRCC1 in BER of our nucleosome substrates is limited in BTNE. In addition, our analysis of L(30)G-L6 nucleosome substrates indicates that SP BER occurs about twice as frequently as LP BER in this substrate compared to the corresponding naked DNA (Fig. 3f). Therefore, the preference for SP BER extends into nucleosome linker DNA.

To further verify the nucleosome-dependent bias for SP BER, we reconstituted the latter steps of BER *in vitro* by incubating the L(15)G-62 substrate with purified Pol β and DNA ligase III. As with BTNEs (Fig. 3d; Supplementary Table S4, 20′ timepoint), the ratio of SP:LP repair products increased over 15-fold when BER was incubated with purified enzymes in nucleosomes as compared to naked DNA (Fig. 3g; Supplementary Table S7, highest [DNA ligase III]). This result corroborates the results obtained with the BTNEs (Fig. 3b–f).

### Polymerase extension is also restricted at single strand gaps in nucleosome cores

The choice between SP and LP repair may be due to how many nucleotides can be extended from the DNA lesion or how quickly the DNA polymerase is able to “handoff” the DNA substrate to DNA ligase^27^. To evaluate these possibilities, we performed the same assay as in Fig. 3 but omitted ATP, allowing the analysis of only extension products. Since ligation is non-functional in the absence of ATP, there cannot be a handoff between DNA polymerase and DNA ligase. Therefore, this assay allows extension of the 1-nt product to a 2-nt product if there are no other impediments for incorporation of a ddNTP. When gaps are located in NCP DNA, the unprocessed substrate (cleaved) or the1-nt extension is predominant over the 2-nt product (Supplementary Fig. S3a–c); however, when the gap is located in linker DNA, the 2-nt extension product is predominant (Supplementary Fig. S3d). Therefore, the NCP restricts BER polymerase extension to 1 nt. Surprisingly, the time course for appearance of the 2-nt extension product in L(30)G-L6 NUC is more rapid than for the naked DNA substrate (Supplementary Fig. S3d).

### Preferential repair of gapped nucleosome substrates by DNA polymerase β

**Figure 4:**
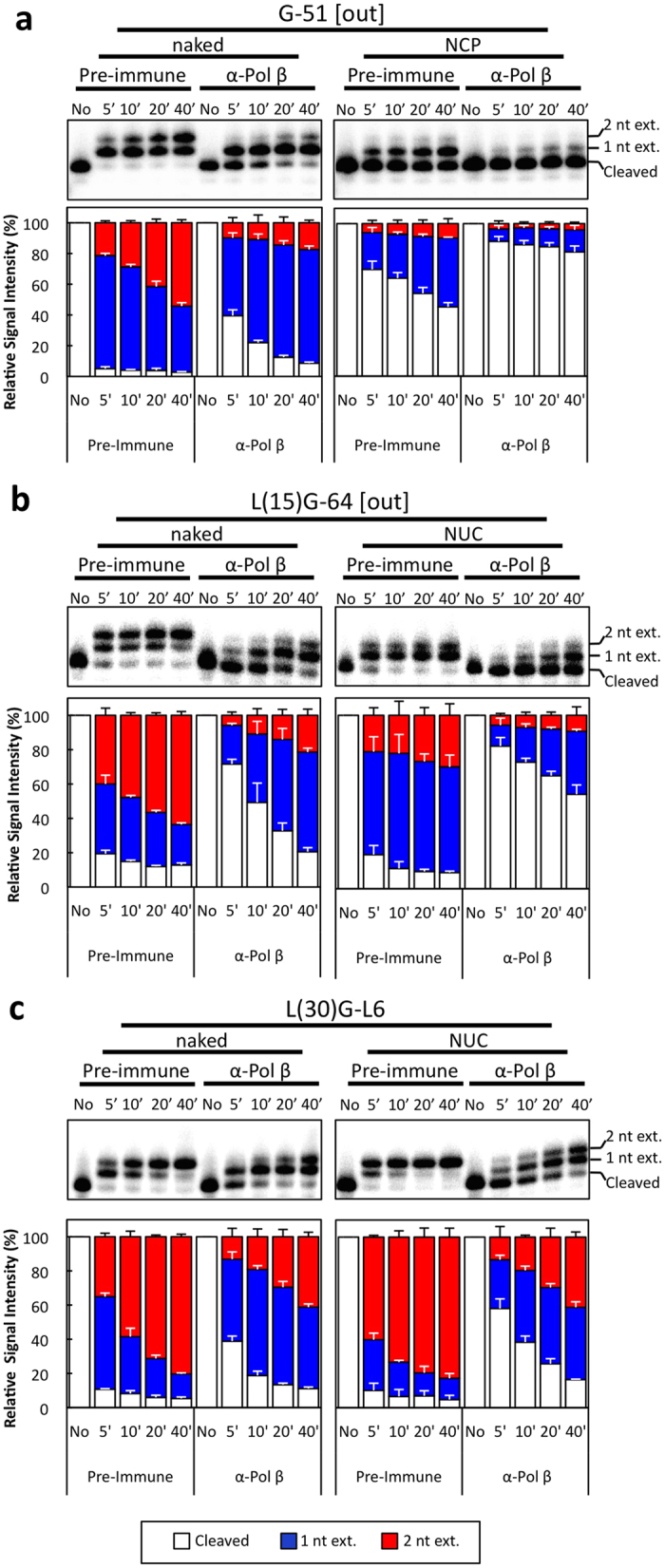
Pol β is important for BER polymerase extension in nucleosome substrates. BTNE was preincubated with either pre-immune or α-Pol β sera for 40 minutes at 0–1 °C and then mixed with (**a**) G-51, (**b**) L(15) G-64, and (**c**) L(30) G-L6 naked and nucleosomal substrates. Composite bar graphs are plotted for each substrate as in Fig. 3 with quantifications in Supplementary Tables S8–10. Standard deviations of 3 replicates from 3 independent nucleosome reconstitutions are shown.

Since BER of nucleosome substrates is clearly biased for SP BER (Fig. 3), which can be performed by Pol β in naked DNA^6^, we hypothesized that BER in nucleosome substrates primarily involves Pol β for extension of BER intermediates. To test this hypothesis, BTNE in the absence of ATP was incubated with polyclonal antibody (α-Pol β) to specifically neutralize Pol β activity^22^ (Fig. 4; Supplementary Fig. S4a and Tables S8–S10). Analyses of G-51 and L(15)-G64 nucleosome substrates treated with α-Pol β serum show ~66% and ~50% decreases, respectively, in BER polymerase extension after 40 minutes as compared to the pre-immune serum control (Fig. 4a,b). This decrease is not solely due to a decline in overall polymerase activity since treatment of naked G-51 and L(15)G-64 DNA substrates with α-Pol β serum allows for ~91% and ~79% polymerase extension after 40 minutes, respectively. However, when the gap is shifted from the NCP to linker DNA, BER polymerase extension only decreases by ~12% at the 40-minute time point for the nucleosome substrate (Fig. 4c), indicating repair in linker DNA is less dependent on Pol β. Pol β is not the only polymerase that is implicated in BER; rather, replicative polymerases δ and ε have been shown to be important for LP BER^7^ and DNA polymerase λ mediates a back-up BER process^28^. Therefore, we treated the extracts with either aphidicolin, an inhibitor of replicative polymerases, or N-ethylmaleimide (NEM), an inhibitor of polymerase λ, in conjunction with anti-Pol β serum. We found that aphidicolin, in agreement with a previous report^22^, and NEM do not affect polymerase extension under our experimental conditions (Supplementary Fig. S4b–c). However, when Pol β-neutralized BTNE was supplemented with only ddTTP, which will incorporate in the 1^st^ position after the gap, there was reduced polymerase extension (Supplementary Fig. S4d). Since Pol β is not inhibited by ddNTPs (Supplementary Fig. S4d, compare lanes 2 & 3; ref. 23), this indicates a ddNTP-sensitive DNA polymerase(s) is present in the nuclear extract. The incorporation of a ddNTP at the 2^nd^ position is important for our SP/LP BER assay (Fig. 3); therefore, we wanted to examine if the ddNTP affects the results of the SP/LP BER assay. As shown in Supplementary Fig. S4e, the substrate can extend up to 3 nts when supplemented with dTTP and dATP because dTTP is the 1^st^ nucleotide incorporated and dATP is the 2^nd^ and 3^rd^ nucleotide incorporated; however, when ddATP is exchanged for dATP, the incorporation stalls at the 2^nd^ nucleotide. Quantification of the extension products beyond 1 nt show that supplementation with dATP or ddATP is similar, indicating that the ddNTP is not dramatically affecting extension past 1 nt in this assay (Supplementary Fig. S4e, compare lanes 2 & 4). These data indicate that 1) when Pol β is neutralized, there are other polymerases that can extend the gapped substrate and 2) ddNTP in the SP/LP BER assay does not affect polymerase extension in BTNEs.

To further verify that Pol β is the preferred polymerase for extension in nucleosomes, we created a nicked DNA substrate that contains a 3′ hydroxyl and 5′ tetrahydrofuran (THF) at position 51, which inhibits the dRPase activity of Pol β and repair patch extension can only proceed through LP BER (Supplementary Fig. S5a)^29^. The extension profile of THF nick-51 naked DNA is similar to the G-51 naked substrate (compare Fig. 4a to Supplementary Fig. S5b). However, for the NCP substrate, the pre-immune extension profile of the THF substrate shows an ~50% decrease in total extension as compared to the G-51 NCP substrate (compare Fig. 4a to Supplementary Fig. S5b). Therefore, without Pol β dRPase activity, extension of an NCP DNA lesion is more restricted. Collectively, these data indicate that Pol β is the primary DNA polymerase responsible for BER of lesions in the NCP.

## Discussion

**Figure 5:**
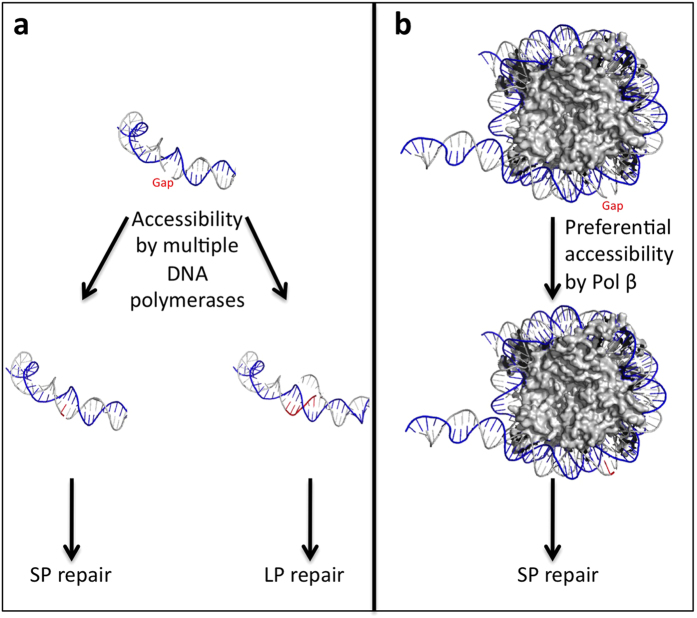
Preferential SP repair of gapped DNA in nucleosomes. (**a**) Naked DNA containing a gap lesion (Gap) is readily repaired in BTNEs via either SP or LP BER. Extended nucleotides are shown in red, and extension in LP BER shows displacement of the DNA strand. (**b**) An equivalent substrate reconstituted in a nucleosome is restricted to a 1 nt extension patch by Pol β thus leading to SP BER. (PDB:1ZBB).

In this study, we identify a novel nucleosome-dependent bias for SP BER when using BTNE (Fig. 3b–f) or purified enzymes (Fig. 3g). In uracil substrates, BER polymerase extension is almost exclusively limited to 1 nt in NCP DNA (Fig. 2c–e). Furthermore, in linker DNA, the extension is halted once the polymerase reaches the NCP (Fig. 2f–g). When using a gap substrate, we determined that this 1-nt limit is imposed by the NCP in the absence of DNA ligase activity (Supplementary Fig. S3a–d), indicating DNA ligase activity is not the limiting factor in nucleotide extension in nucleosomes. Furthermore, this 1-nt limit within NCPs will inhibit additional extension that can be performed by Pol β and flap endonuclease via a “Hit and Run” mechanism^10^. Additional extension in the nucleosome can be forced when the concentration of DNA ligase is in excess as compared to the DNA polymerase (Fig. 3g); however, 2-nt polymerase extension is reduced in nucleosomes as compared to naked DNA corroborating the data shown with BTNEs (Supplementary Fig. S3a–d). These results indicate that BER of DNA lesions in NCPs is strongly biased for SP BER as summarized in Fig. 5.

Previous studies analyzed purified glycosylase cleavage activity in linker DNA (±histone H1) of mono- or di-nucleosome substrates^16^,^25^ and found that, in the absence of H1, DNA outside the NCP region exhibits cleavage efficiencies similar to free DNA. Our data show that glycosylase cleavage and BER synthesis activities in BTNEs are partially inhibited in linker DNA, particularly when the lesion is near the NCP (Fig. 2). Since nucleosomes with uracil positioned in linker DNA have reduced cleavage in BTNEs (Fig. 2f–h), it can be posited that nucleosomes inherently inhibit overall cleavage. However, we do not think this is the case since cleavage of L(15)U-62 NUC and its corresponding naked DNA substrate are very similar (Fig. 2d). Therefore, reduced repair in linker DNA may be due to transient DNA “unwrapping” that occurs at the entry/exit site of NCPs^30^,^31^ (compare U-51[out] and L(15)U-62[out] in Fig. 2b,d) and may extend into linker DNA (Fig. 2f–h) because DNA linker-histone contacts may occlude repair enzymes at these sites. We hypothesize that DNA unwrapping affects linker DNA repair and the nearby NCP adds steric hindrance to repair-associated factors. Additionally, to our knowledge, this is the first study to show that DNA synthesis during BER can only progress 2 nt into the NCP (Fig. 2f–g).

To investigate the role of Pol β SP BER of NCPs, we selectively inhibited Pol β activity in the extract with a neutralizing antibody (i.e., α-Pol β serum, Fig. 4). Since there is still polymerase activity after Pol β neutralization, this indicates that there is Pol β-independent extension of the DNA substrates. Although mammals contain multiple DNA polymerases for BER (Pol β, δ, ε, γ, θ, ι, and λ^7^,^32^,^33^,^34^,^35^,^36^), under our conditions, BER extension in BTNEs is independent of Pol δ, ε and λ (Supplementary Fig. S4b,c). Instead, BTNE polymerase extension is sensitive to ddTTP (Supplementary Fig. S4d, compare lanes 5 & 7). Pol γ^37^ and θ^23^,^38^ have been shown to be sensitive to ddNTPs, and we believe that Pol θ is most likely the major ddNTP-sensitive polymerase present in BTNE because Pol γ is a mitochondrial polymerase^39^ and Pol θ transcripts are highly expressed in mammalian testes^38^.

Alternatively, our Pol β-neutralization results indicate that Pol β is responsible for the majority of BER synthesis at gapped substrates in NCP DNA (Fig. 4), while substantial DNA polymerase activity is maintained in naked DNA when Pol β is neutralized. Interestingly, even though the uracil in L(30)G-L6 NUC is located in linker DNA, there is still a preference for SP BER in the nucleosome substrate as compared to the naked DNA substrate (Fig. 3f). Furthermore, the same substrate showed a faster accumulation of 2-nt extension products in the nucleosome substrate as compared to the naked DNA (Supplementary Fig. S3d). Since our data suggest Pol β is the main polymerase to access lesions at position −51 (Fig. 4a), it seems likely that Pol β can access THF nick lesions in the NCP substrate. Interestingly, Pol β cannot process the THF nick as efficiently as the gapped substrate in the NCP (Fig. 4a and Supplementary Fig. S4, compare Pre-immune/NCPs), which is most likely due to the inability of Pol β to process THF nicks as efficiently as gapped substrates^10^. This provides further evidence that the extracts contain other polymerases to process THF nicks (Supplementary Fig. S5B; compare naked to NCP). Collectively, these results suggest there is a selection for the DNA polymerase that carries out BER of gapped substrates at or near the NCP-linker junction (Fig. 5). Pol β is the smallest mammalian DNA polymerase at 38 kDa^32^; therefore, we hypothesize steric hindrance excludes bulkier polymerases (*e.g.,* the 290 kDa Pol θ) from extending gap lesions in NCP DNA. Indeed, as Pol β has been shown to be evolutionarily linked to the development of metazoans^40^, it is possible that this DNA polymerase evolved to repair compact DNA because it is less restricted in a compact chromatin environment than other DNA polymerases.

## Methods

### DNA and nucleosome substrates

PCR amplicons entailing the 601 nucleosome positioning sequence and their accompanying primers are described in Supplementary Table S1. See Supplementary Experimental Procedures for details. Nucleosome reconstitutions were performed by salt dialysis of equimolar amounts of recombinant *Xenopus laevis* histone octamers (gift from Dr. Ming-Rui Duan) and DNA substrates^41^ followed by analysis on 6% native polyacrylamide gels.

### Hydroxyl radical footprinting

Naked and nucleosome DNAs were treated with hydroxyl radicals (•OH) as previously described^14^,^15^. See Supplementary Experimental Procedures for details.

### Repair reactions

To analyze BER polymerase extension and cleavage activity in naked and nucleosome DNAs that contain a uracil, 50 nM of DNA was incubated in 50 mM HEPES pH 7.5, 0.5 mM EDTA pH 8.0, 2 mM DTT, 25 mM KCl, 10 mM MgCl_2_, 10 μM ssDNA 19 mer, 0.1 mM of all four dNTPs, and 13 μg of bovine testis nuclear extract (BTNE) in a 10 μL reaction mix. The samples were treated at 37° for the indicated times and then resuspended in formamide loading buffer (50% formamide and 10 mM EDTA). This was then mixed with 0.1 units of proteinase K (Fermentas) and incubated at 55 °C for 15 minutes to digest protein and subsequently incubated at 95 °C for 5 minutes to denature DNA. The samples were run at 60 watts on an 8% urea sequencing gel casted in a 21 × 50 cm Sequi-Gen apparatus (Bio-Rad) for 1.25 hours to resolve cleaved and extension products and subsequently dried with a gel dryer (Bio-Rad). The gels were exposed to a phosphor screen, scanned via Typhoon FLA 7000 (GE Healthcare Life Sciences), and analyzed by ImageQuant TL (GE Healthcare Life Sciences). See Supplementary Experimental Procedures for details on other repair reactions.

## Additional Information

**How to cite this article**: Meas, R. and Smerdon, M. J. Nucleosomes determine their own patch size in base excision repair. *Sci. Rep.* **6**, 27122; doi: 10.1038/srep27122 (2016).

## Supplementary Material

Supplementary Information
