# Supplementary material for: Nucleosomes determine their own patch size in base excision repair
Source: Sci Rep. 2016 Jun 6;6:27122. doi: 10.1038/srep27122 (PMC4893620; doi:10.1038/srep27122)
Supplement: Supplementary Information [file srep27122-s1.pdf]

Nucleosomes determine their own patch size in base excision repair

Rithy Meas and Michael J. Smerdon
School of Molecular Biosciences
Washington State University
Pullman, WA 99164-7520

<u>Supplementary figures</u>

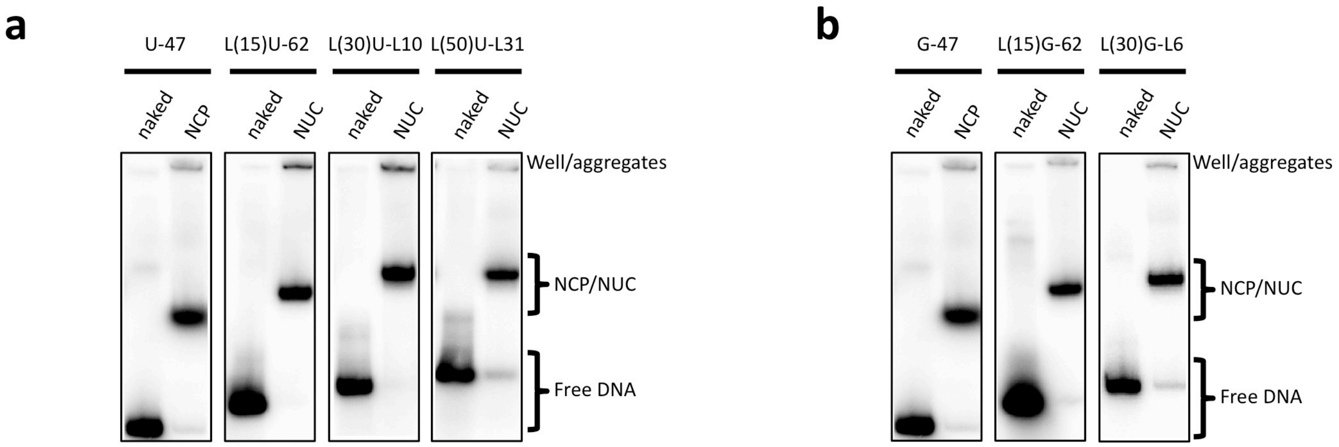

Supplementary Figure S1. Nucleosome reconstitutions of uracil and gap substrates. ^{32}P-end labeled **A**) uracil and **B**) gapped substrates were reconstituted by salt dialysis [1] and ran on a 6% native acrylamide gel.

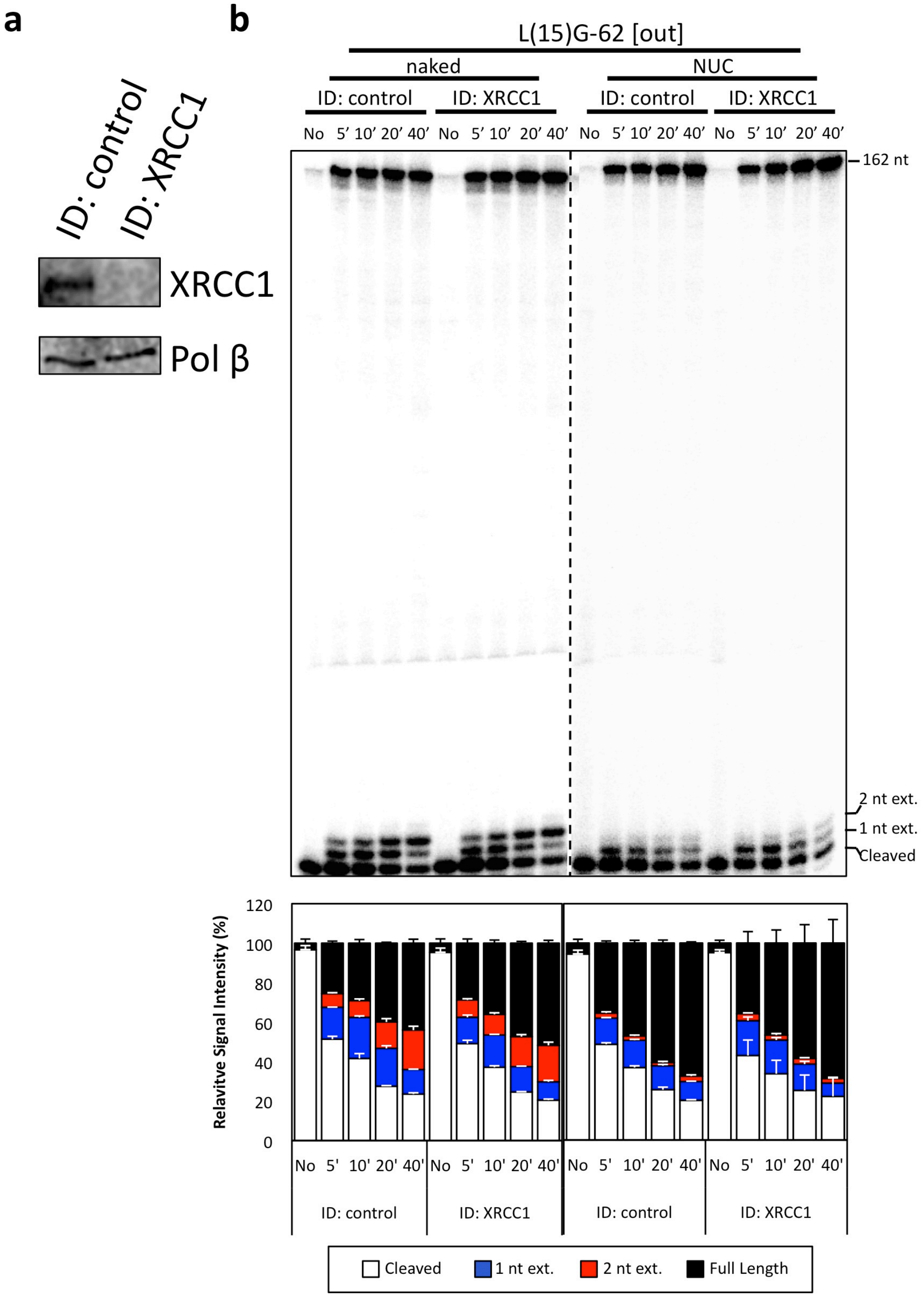

Supplementary Figure S2. Immunodepletion of XRCC1 from BTNE does not affect SP BER preference in nucleosome substrates. A) Western blot analysis was conducted with mock-immunodepleted (ID: control) and ID: XRCC1 BTNE. Pol β is used as a loading control. **B**) BER cleavage/extension assay was performed (as **Figure 2)** with L(15)G-62 naked and nucleosome substrates in the presence of ID: control or ID: XRCC1 BTNE. Composite bar graphs (white, cleaved; red, 1 nt; blue, 2nt; and black, full length) are plotted. The standard deviations of 3 replicates from 3 independent nucleosome reconstitutions are shown.

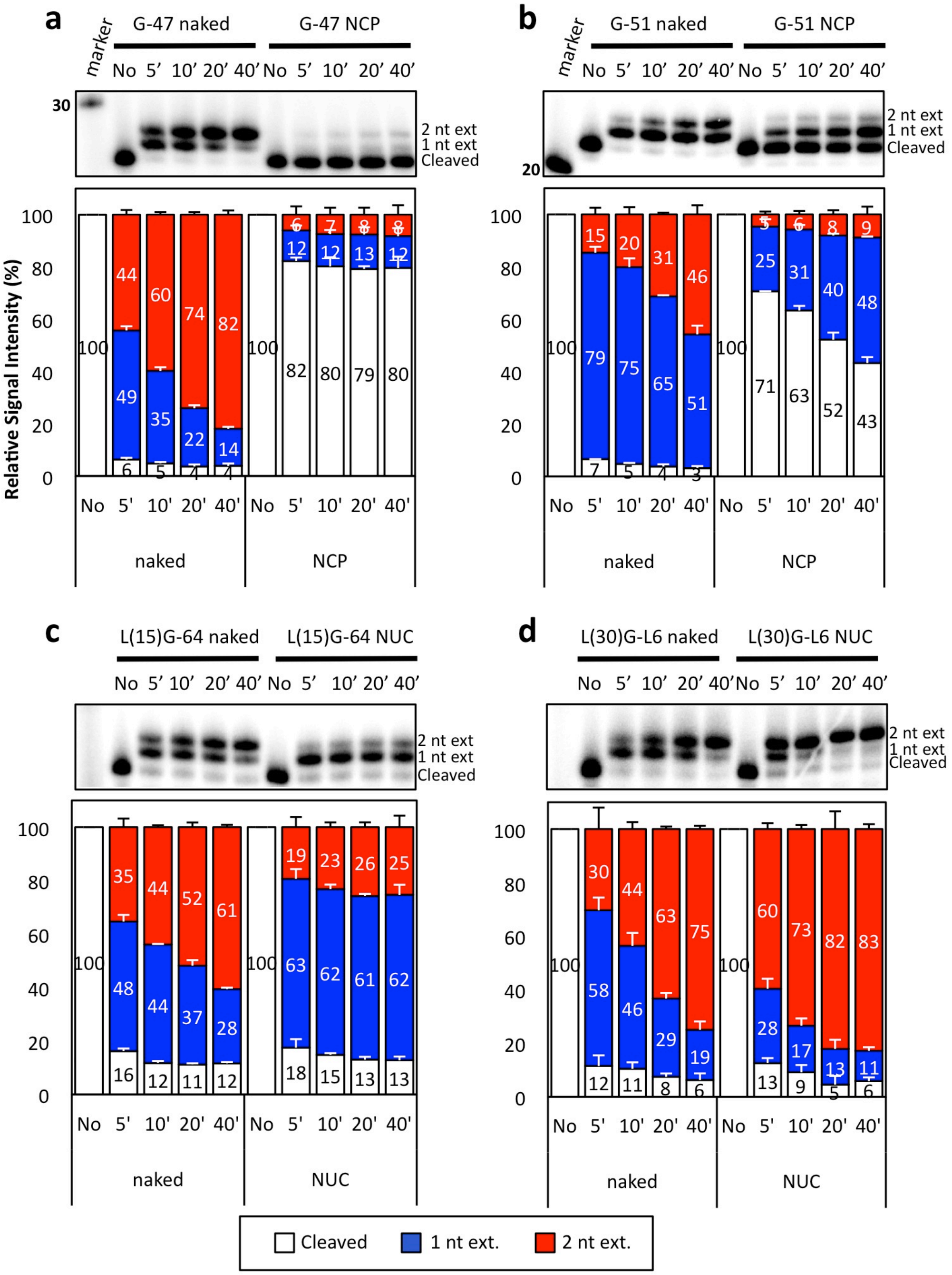

Supplementary Figure S3. BER polymerase extension is restricted in nucleosome core DNA and is independent of replicative polymerases. Gap substrates **A**) G-47, **B**) G-51, **C**) L(15)G-64, and **D**) L(30)-L6 were incubated in BTNE, ddNTP, dTTP, in the absence of ATP and run on denaturing sequencing gels. BER polymerase can extend up to 2 nt at the gap substrate through the incorporation of a ddNTP. Due to minimal ligase activity, the full-length band is constant among samples (not shown). **E**) Pol β neutralization was performed (as **Figure 4**) on the L(15)G-64 substrate with or without aphidicolin (500 μg/mL) followed by a 37°C incubation for 20 minutes. Composite bar graphs containing the quantifications of the relative signal intensities of the three DNA bands (white, cleaved; red, 1 nt; and blue, 2 nt) are shown representing at least 3 replicates.

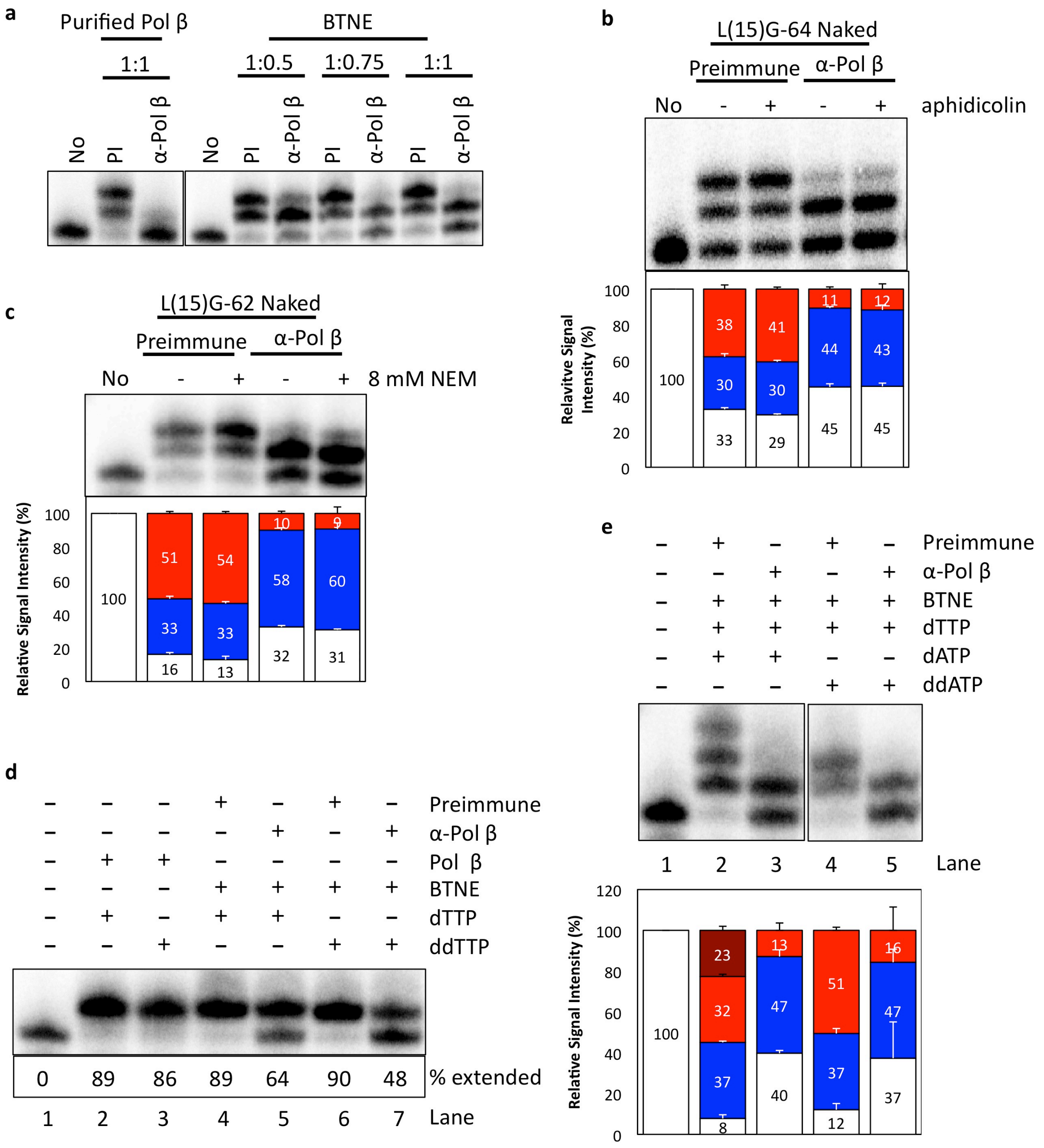

Supplementary Figure S4. BTNE contains Pol β and ddNTP-sensitive polymerase(s). a) Purified Pol β (12 nM) or BTNE was pre-incubated with preimmune (PI) or α-Pol β sera for 40 minutes at a ratio of 1: 05,0.75, or 1 purified Pol β/BTNE:serum followed by incubation with L(15)G-62 DNA for 10 minutes at 37°C. All subsequent assays (**b-e**) were performed with a 1:1 ratio of BTNE:serum. **b)** Pol β neutralization of BTNE was performed on the L(15)G-64 DNA substrate with or without aphidicolin (500 µg/mL) followed by a 37°C incubation for 20 minutes. **c)** Pre immune or Pol β-neutralized BTNE was treated with or without 8 mM NEM and incubated with L(15)G-62 DNA at 37°C for 10 minutes. **d)** Pol β (12 nM), BTNE, or Pol β-neutralized BTNE was incubated with L(15)G-62 DNA and supplemented with either dTTP or ddTTP as the only nucleotide at 37°C for 10 minutes. Purified Pol β was used as control for incorporation of ddTTP. The % extended is shown below the phosphorimager scan. **e)** Pre-immune or Pol β-neutralized BTNE was incubated with L(15)G-62 DNA that was supplemented with dTTP and either dATP or ddATP. Extension can proceed to three nucleotides because both the 2nd and 3rd complementary bases are thymidines. Composite bar graphs of the relative signal intensities of the DNA bands (white, cleaved; blue, 1 nt extension; red, 2 nt extension; and burgundy, 3 nt extension) are shown representing at least 3 replicates.

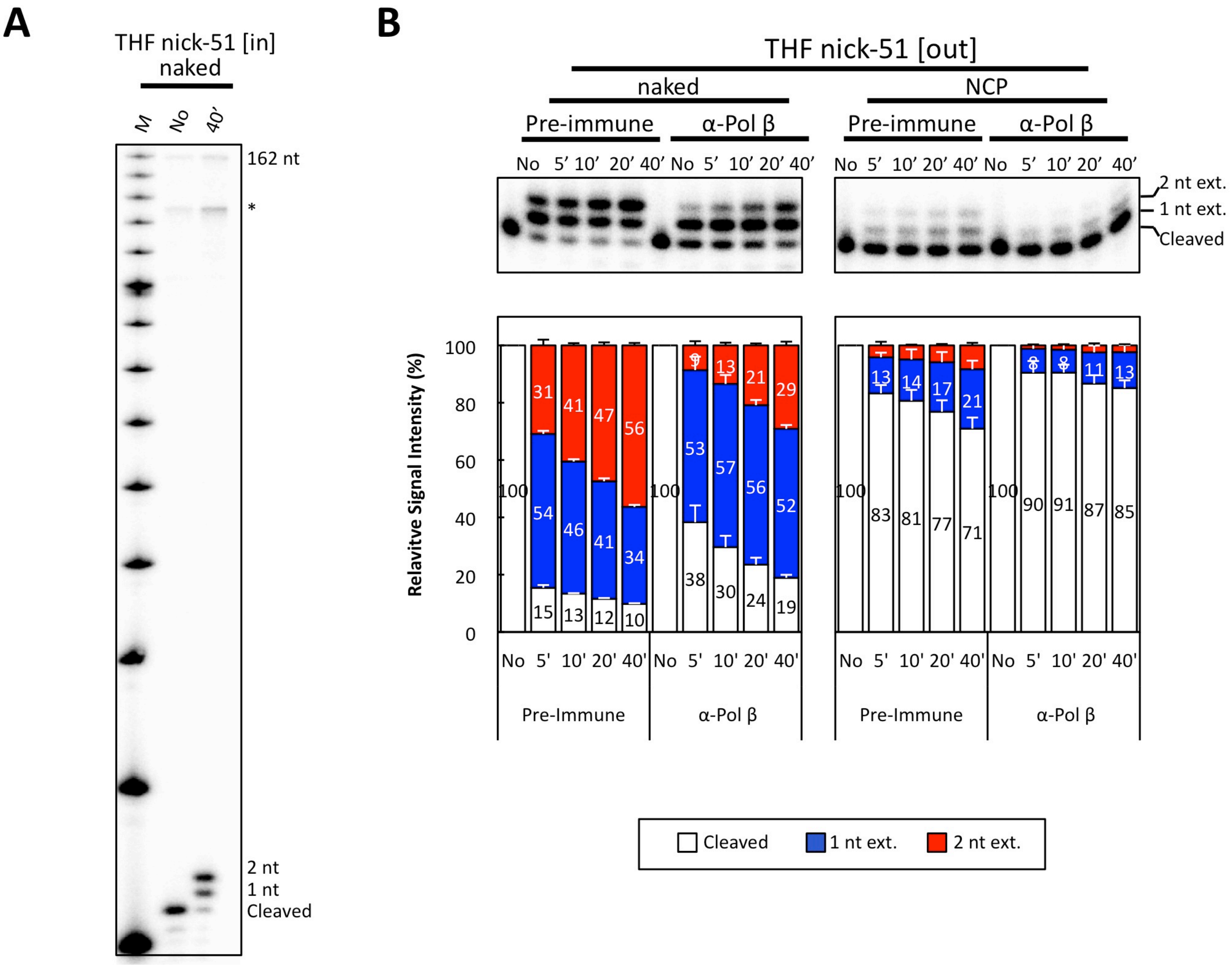

Supplementary Figure S5. The THF nick-51 substrate shows reduced SP BER and polymerase extension activity. A) SP/LP assay (as **Figure 3**) products using the THF nick-51 substrate were run on a denaturing sequencing gel (* minor PCR product). **B**) Pol β neutralization was conducted (as **Figure 4)** for the THF nick-51 substrate. Composite bar graphs of the relative signal intensities of the three DNA bands (white, cleaved; red, 1 nt; and blue, 2 nt) are shown.

Supplementary Table S1. DNA substrates used.

DNA substrate	DNA sequence[1,2,3]	Primers
601-147+L50<br>undamaged	GCTCGGAACACTATCCGACTGGCACCGGCAAGGTCGCTGT<br>TCAATACATGCAGGATGTATATATCTGACACGTGCCTGGA<br>GACTAGGGAGTAATCCCCTTGGCGGTTAAAACGCGGGGGA<br>CAGCGCGTACGTGCGTTTAAGCGGTGCTAGAGCTGTCTAC<br>GACCAATTGAGCGGCCTCGGCACCGGGATTCTCCAGG	5'GGCACCGGCAAGGTCGC<br>TGTTCAATACATGCA<br>5'/Biot/CCT GGA GAA TCC<br>CGG TGC CGA GGC
601-147<br>U-47	CAGGATGTATATATCTGACACGTGCCUGGAGACTAGGGAG<br>TAATCCCCTTGGCGGTTAAAACGCGGGGGACAGCGCGTAC<br>GTGCGTTTAAGCGGTGCTAGAGCTGTCTACGACCAATTGA<br>GCGGCCTCGGCACCGGGATTCTCCAGG	5'CAGGATGTATATATCTGA<br>CACGTGCCUGGAGA<br>5'/Biot/CCT GGA GAA TCC<br>CGG TGC CGA GGC
601-147<br>U-51	CAGGATGTATATATCTGACACGUGCCTGGAGACTAGGGAG<br>TAATCCCCTTGGCGGTTAAAACGCGGGGGACAGCGCGTAC<br>GTGCGTTTAAGCGGTGCTAGAGCTGTCTACGACCAATTGA<br>GCGGCCTCGGCACCGGGATTCTCCAGG	5'CAGGATGTATATATCTGA<br>CACGUGCCTGGAGA<br>5'/Biot/CCT GGA GAA TCC<br>CGG TGC CGA GGC
601-147<br>THF-51	CAGGATGTATATATCTGACACGTHFGCCTGGAGACTAGGG<br>AGTAATCCCCTTGGCGGTTAAAACGCGGGGGACAGCGCGT<br>ACGTGCGTTTAAGCGGTGCTAGAGCTGTCTACGACCAATT<br>GAGCGGCCTCGGCACCGGGATTCTCCAGG	5'CAGGATGTATATATCTGA<br>CACGTHFGCCTGGAGA<br>5'/Biot/CCT GGA GAA TCC<br>CGG TGC CGA GGC
601-147+L15<br>L(15)U-62	GCTGTTCAATACATGCAGGATGTATAUATCTGACACGTGC<br>CTGGAGACTAGGGAGTAATCCCCTTGGCGGTTAAAACGCG<br>GGGGACAGCGCGTACGTGCGTTTAAGCGGTGCTAGAGCTG<br>TCTACGACCAATTGAGCGGCCTCGGCACCGGGATTCTCCA<br>GG	5'GCTGTTCAATACATGCAG<br>GATGTATAUATCTG<br>5'/Biot/CCT GGA GAA TCC<br>CGG TGC CGA GGC
601-147+L15<br>L(15)U-64	GCTGTTCAATACATGCAGGATGTAUATATCTGACACGTGC<br>CTGGAGACTAGGGAGTAATCCCCTTGGCGGTTAAAACGCG<br>GGGGACAGCGCGTACGTGCGTTTAAGCGGTGCTAGAGCTG<br>TCTACGACCAATTGAGCGGCCTCGGCACCGGGATTCTCCA<br>GG	5'GCTGTTCAATACATGCAG<br>GATGTAUATATCTG<br>5'/Biot/CCT GGA GAA TCC<br>CGG TGC CGA GGC
601-147+L30<br>L(30)U-L6	GGCACCGGCAAGGTCGCTGTTCAAUACATGCAGGATGTAT<br>ATATCTGACACGTGCCTGGAGACTAGGGAGTAATCCCCTT<br>GGCGGTTAAAACGCGGGGGACAGCGCGTACGTGCGTTTAA<br>GCGGTGCTAGAGCTGTCTACGACCAATTGAGCGGCCTCGG<br>CACCGGGATTCTCCAGG	5'GGCACCGGCAAGGTCGC<br>TGTTCAAUACATGCA<br>5'/Biot/CCT GGA GAA TCC<br>CGG TGC CGA GGC
601-147+L30<br>L(30)U-L10	GGCACCGGCAAGGTCGCTGTUCAATACATGCAGGATGTAT<br>ATATCTGACACGTGCCTGGAGACTAGGGAGTAATCCCCTT<br>GGCGGTTAAAACGCGGGGGACAGCGCGTACGTGCGTTTAA<br>GCGGTGCTAGAGCTGTCTACGACCAATTGAGCGGCCTCGG<br>CACCGGGATTCTCCAGG	5'GGCACCGGCAAGGTCGC<br>TGTUCAATACATGCA<br>5'/Biot/CCT GGA GAA TCC<br>CGG TGC CGA GGC
601-147+L50<br>L(50)U-L31	GCTCGGAACACTATCCGACUGGCACCGGCAAGGTCGCTGT<br>TCAATACATGCAGGATGTATATATCTGACACGTGCCTGGA<br>GACTAGGGAGTAATCCCCTTGGCGGTTAAAACGCGGGGGA<br>CAGCGCGTACGTGCGTTTAAGCGGTGCTAGAGCTGTCTAC<br>GACCAATTGAGCGGCCTCGGCACCGGGATTCTCCAGG	5'GCTCGGAACACTATCCGA<br>CUGGCACCGGCAAG<br>5'/Biot/CCT GGA GAA TCC<br>CGG TGC CGA GGC

[1]The base at the nucleosome dyad is denoted as C
[2]Linker DNA is underlined
[3]Uracils denoted as U
[4]Tetrahydrofuran denoted as THF

Supplemental Table *S*2: Quantifications for Figure 3B

G-47					
Relative signal intensity (%) ± S.D.					
		**Cleaved**	**1 nt ext.**	**2 nt ext.**	**Full length**
**Naked**	**No**	95.4 ± 1.9	0 ± 0	0 ± 0	4.6 ± 1.9
	**5'**	7.1 ± 1.0	32.8 ± 3.0	39.3 ± 3.9	20.8 ± 0.4
	**10'**	4.4 ± 0.8	23.6 ± 6.0	44.4 ± 4.6	27.6 ± 1.6
	**20'**	3.8 ± 1.1	11.6 ± 1.8	47.0 ± 3.7	37.6 ± 3.2
	**40'**	4.5 ± 1.9	7.9 ± 2.3	49.3 ± 6.4	38.3 ± 4.9
**NCP**	**No**	97.2 ± 0.3	0 ± 0	0 ± 0	2.8 ± 0.3
	**5'**	76.8 ± 8.0	7.7 ± 4.7	5.6 ± 2.2	9.9 ± 2.9
	**10'**	75.5 ± 1.2	9.0 ± 3.7	5.5 ± 2.7	10 ± 1.7
	**20'**	74.1 ± 5.6	11.3 ± 2.3	5.3 ± 2.5	9.3 ±1.9
	**40'**	70.6 ± 4.0	12.6 ± 0.9	5.8 ± 2.2	11.0 ± 2.6

Supplemental Table *S*3: Quantifications for Figure 3C

G-51					
Relative signal intensity (%) ± S.D.					
		**Cleaved**	**1 nt ext.**	**2 nt ext.**	**Full length**
**Naked**	**No**	94.6 ± 1.0	0 ± 0	0 ± 0	5.4 ± 1.0
	**5'**	7.0 ± 1.0	52.7 ± 3.1	12.5 ± 2.0	27.8 ± 2.5
	**10'**	4.4 ± 1.1	35.4 ± 4.5	15.7 ± 1.8	44.5 ± 5.3
	**20'**	3.2 ± 0.5	11.2 ± 3.1	18.5 ± 0.6	67.0 ± 3.2
	**40'**	2.2 ± 0.8	5.8.1.5	18.8 ± 2.0	73.1 ± 0.3
**NUC**	**No**	94.6 ± 1.0	0 ± 0	0 ± 0	5.4 ± 1.0
	**5'**	65.8 ± 0.8	16.3 ± 2.3	3.6 ± 0.4	14.3 ± 2.4
	**10'**	58.5 ± 0.4	20.1 ± 3.0	4.3 ± 0.3	17.1 ± 2.6
	**20'**	48.4 ± 1.3	28.7 ± 2.0	4.6 ± 0.4	18.3 ± 1.3
	**40'**	39.7 ± 1.3	37.7 ± 2.2	4.6 ± 0.4	18.0 ± 1.3

Supplemental Table *S*4: Quantifications for Figure 3D

L(15)G-62					
Relative signal intensity (%) ± S.D.					
		**Cleaved**	**1 nt ext.**	**2 nt ext.**	**Full length**
**Naked**	**No**	85.7 ± 5.2	0 ± 0	0 ± 0	14.3 ± 5.2
	**5'**	12.0 ± 3.0	31.5 ± 3.1	28.1 ± 3.3	28.4 ± 4.0
	**10'**	8.4 ± 1.3	20.8 ± 1.8	35.1 ± 4.7	35.6 ± 5.5
	**20'**	6.7 ± 0.7	11.9 ± 1.0	44.0 ± 2.0	37.4 ± 2.0
	**40'**	6.6 ± 1.7	7.5 ± 0.5	47.5 ± 2.5	38.4 ± 4.0
**NUC**	**No**	89.6 ± 3.6	0 ± 0	0 ± 0	10.4 ± 3.6
	**5'**	41.4 ± 10.6	10.8 ± 3.2	4.9 ± 1.2	42.9 ± 7.7
	**10'**	22.8 ± 8.6	8.7 ± 0.5	5.9 ± 1.7	62.6 ± 9.6
	**20'**	10.8 ± 6.3	8.8 ± 2.5	4.8 ± 2.0	75.6 ± 9.9
	**40'**	7.5 ± 5.0	9.0 ± 3.8	5.2 ± 2.1	78.2 ± 10.7

Supplemental Table *S*5: Quantifications for Figure 3E

L(15)G-64					
Relative signal intensity (%) ± S.D.					
		**Cleaved**	**1 nt ext.**	**2 nt ext.**	**Full length**
**Naked**	**No**	88.6 ± 1.1	0 ± 0	0 ± 0	11.4 ± 1.1
	**5'**	13.8 ± 1.0	28.2 ± 1.1	27.3 ± 1.1	30.7 ± 2.9
	**10'**	10.6 ± 0.6	21.7 ± 2.1	33.1 ± 1.9	34.7 ± 3.1
	**20'**	8.9 ± 0.3	14.6 ± 0.6	38.2 ± 2.2	38.3 ± 3.0
	**40'**	8.4 ± 0.6	9.4 ± 1.4	41.8 ± 2.7	40.4 ± 3.0
**NUC**	**No**	87.6 ± 1.6	0 ± 0	0 ± 0	12.4 ± 1.6
	**5'**	14.0 ± 2.9	12.0 ± 5.7	8.2 ± 3.4	65.8 ± 11.9
	**10'**	9.0 ± 1.6	7.6 ± 3.0	8.4 ± 4.1	75.0 ± 8.5
	**20'**	7.5 ± 0.7	5.3 ± 0.6	9.2 ± 3.5	78.1 ± 3.3
	**40'**	6.7 ± 1.0	5.1 ± 0.2	10.6 ± 3.6	77.6 ± 3.2

Supplemental Table *S*6: Quantifications for Figure 3F

L(30)G-L6					
Relative signal intensity (%) ± S.D.					
		**Cleaved**	**1 nt ext.**	**2 nt ext.**	**Full length**
**Naked**	**No**	88.2 ± 1.3	0 ± 0	0 ± 0	11.8 ± 1.3
	**5'**	11.3 ± 6.2	30.4 ± 7.7	26.4 ± 0.5	32.0 ± 3.5
	**10'**	6.7 ± 2.6	17.2 ± 5.2	36.1 ± 2.7	40.0 ± 5.1
	**20'**	5.0 ± 1.7	9.5 ± 2.3	40.9 ± 4.6	44.5 ± 5.7
	**40'**	4.8 ± 2.8	6.9 ± 0.6	42.6 ± 5.0	45.7 ± 4.9
**NUC**	**No**	87.5 ± 1.9	0 ± 0	0 ± 0	12.5 ± 1.9
	**5'**	18.1 ± 2.3	4.8 ± 0.8	22.3 ± 1.1	54.8 ± 2.7
	**10'**	13.9 ± 1.9	5.2 ± 1.1	23.9 ± 2.0	57.0 ± 2.6
	**20'**	12.7 ± 2.0	5.1 ± 1.8	25.0 ± 3.1	57.2 ± 2.1
	**40'**	11.6 ± 1.8	5.2 ± 1.4	27.1 ± 4.8	56.1 ± 4.6

Supplemental Table *S*7: Quantifications for Figure 3G

L(15)G-62					
Relative signal intensity (%) ± S.D.					
		**Cleaved**	**1 nt ext.**	**2 nt ext.**	**Full length**
**Naked**	**No**	94.7 ± 0.8	0 ± 0	0 ± 0	5.3 ± 0.81
	**18.5**	2.0 ± 0.3	1.5 ± 0.2	72.1 ± 3.3	24.4 ± 3.3
	**37**	1.5 ± 0.1	1.2 ± 0.1	71.6 ± 1.9	25.7 ± 2.1
	**74**	1.5 ± 0.2	1.4 ± 0.2	65.9 ± 1.0	31.2 ± 1.1
	**147**	1.4 ± 0.2	1.2 ± 0.7	60.0 ± 1.5	37.6 ± 0.8
	**294**	2.9 ± 1.0	1.9 ± 0.5	50.0 ± 1.1	45.2 ± 1.1
**NUC**	**No**	94.3 ± 1.0	0 ± 0	0 ± 0	5.7 ± 1.0
	**18.5**	10.9 ± 1.5	33.7 ± 0.1	35.9 ± 2.6	19.5 ± 2.3
	**37**	11.7 ± 0.9	21.1 ± 1.9	22.4 ± 2.1	44.8 ± 4.1
	**74**	4.1 ± 0.7	7.9 ± 2.0	10.6 ± 1.8	77.4 ± 4.4
	**147**	5.6 ± 1.1	6.0 ± 1.8	4.8 ± 0.8	83.6 ± 3.6
	**294**	5.4 ± 1.3	4.4 ± 1.4	3.2 ± 1.0	87.0 ± 3.6

Supplemental Table *S*8: Quantifications for Figure 4a

G-51 naked				
Relative signal intensity (%) ± S.D.				
		**Cleaved**	**1 nt ext.**	**2 nt ext.**
**Preimmune**	**No**	100 ± 0	0 ± 0	0 ± 0
	**5′**	5.0 ± 1.3	73.6 ± 1.5	21.4 ± 1.3
	**10′**	4.0 ± 0.5	67.2 ± 1.7	28.8 ± 1.4
	**20′**	3.9 ± 1.4	54.6 ± 3.4	41.5 ± 2.4
	**40′**	2.8 ± 0.3	42.9 ± 2.2	54.3 ± 2.0
**α-Pol β**	**No**	100 ± 0	0 ± 0	0 ± 0
	**5′**	39.6 ± 3.8	50.6 ± 3.3	9.8 ± 3.4
	**10′**	22.0 ± 1.6	67.2 ± 3.5	10.9 ± 5.1
	**20′**	12.5 ± 1.2	73.1 ± 2.7	14.4 ± 3.8
	**40′**	8.6 ± 0.7	74.0 ± 2.3	17.4 ± 1.7

G-51 NCP				
Relative signal intensity (%) ± S.D.				
		**Cleaved**	**1 nt ext.**	**2 nt ext.**
**Preimmune**	**No**	100 ± 0	0 ± 0	0 ± 0
	**5′**	70.0 ± 5.5	24.2 ± 3.3	5.9 ± 2.1
	**10′**	64.2 ± 3.7	28.8 ± 1.5	7.0 ± 2.3
	**20′**	54.2 ± 3.9	37.3 ± 1.6	8.5 ± 2.4
	**40′**	45.4 ± 2.8	45.1 ± 0.6	9.4 ± 3.3
**α-Pol β**	**No**	100 ± 0	0 ± 0	0 ± 0
	**5′**	88.4 ± 3.2	8.1 ± 2.3	3.5 ± 1.8
	**10′**	86.1 ± 2.8	11.0 ± 1.7	2.8 ± 1.1
	**20′**	84.9 ± 2.6	11.6 ± 1.5	3.5 ± 1.2
	**40′**	81.5 ± 3.9	14.5 ± 2.9	4.1 ± 0.9

Supplemental Table *S*9: Quantifications for Figure 4b

L(15)G-64 naked				
Relative signal intensity (%) ± S.D.				
		**Cleaved**	**1 nt ext.**	**2 nt ext.**
**Preimmune**	**No**	100 ± 0	0 ± 0	0 ± 0
	**5′**	19.4 ± 2.0	40.6 ± 5.1	40.0 ± 4.2
	**10′**	14.9 ± 0.8	37.3 ± 1.2	47.9 ± 1.6
	**20′**	12.0 ± 0.6	31.5 ± 1.2	56.6 ± 1.5
	**40′**	12.8 ± 1.2	23.6 ± 1.0	63.6 ± 2.2
**α-Pol β**	**No**	100 ± 0	0 ± 0	0 ± 0
	**5′**	71.6 ± 2.8	22.6 ± 1.1	5.8 ± 1.9
	**10′**	49.3 ± 11.2	39.8 ± 7.4	10.9 ± 3.9
	**20′**	32.7 ± 4.5	53.2 ± 6.5	14.1 ± 3.5
	**40′**	20.5 ± 2.4	58.1 ± 2.3	21.4 ± 3.6

L(15)G-64 NUC				
Relative signal intensity (%) ± S.D.				
		**Cleaved**	**1 nt ext.**	**2 nt ext.**
**Preimmune**	**No**	100 ± 0	0 ± 0	0 ± 0
	**5′**	19.7 ± 5.3	59.3 ± 8.6	21.0 ± 4.4
	**10′**	11.7 ± 3.9	66.4 ± 10.9	22.0 ± 8.1
	**20′**	9.9 ± 1.3	63.4 ± 4.4	26.7 ± 4.5
	**40′**	9.3 ± 0.9	61.0 ± 6.8	29.7 ± 6.7
**α-Pol β**	**No**	100 ± 0	0 ± 0	0 ± 0
	**5′**	82.3 ± 4.9	12.0 ± 4.0	5.7 ± 1.1
	**10′**	73.0 ± 2.2	20.0 ± 2.1	7.0 ± 1.8
	**20′**	65.2 ± 2.6	26.9 ± 1.1	7.9 ± 1.8
	**40′**	54.5 ± 5.4	36.3 ± 1.2	9.2 ± 4.9

Supplemental Table *S*10: Quantifications for Figure 4c

L(30)G-6 naked				
Relative signal intensity (%) ± S.D.				
		**Cleaved**	**1 nt ext.**	**2 nt ext.**
**Preimmune**	**No**	100 ± 0	0 ± 0	0 ± 0
	**5′**	10.8 ± 0.3	54.2 ± 2.1	35.1 ± 2.1
	**10′**	8.2 ± 1.7	33.3 ± 4.9	58.5 ± 3.3
	**20′**	6.0 ± 1.3	22.8 ± 1.9	71.3 ± 1.0
	**40′**	5.3 ± 0.9	14.3 ± 0.7	80.3 ± 1.5
**α-Pol β**	**No**	100 ± 0	0 ± 0	0 ± 0
	**5′**	38.9 ± 3.0	48.0 ± 4.3	13.1 ± 4.9
	**10′**	18.8 ± 2.5	62.1 ± 2.2	19.2 ± 4.6
	**20′**	13.4 ± 0.9	57.1 ± 3.4	29.6 ± 4.2
	**40′**	11.1 ± 0.9	47.7 ± 1.9	41.2 ± 2.6

GL(30)-6 NUC				
Relative signal intensity (%) ± S.D.				
		**Cleaved**	**1 nt ext.**	**2 nt ext.**
**Preimmune**	**No**	100 ± 0	0 ± 0	0 ± 0
	**5′**	10.2 ± 4.2	29.7 ± 3.8	60.1 ± 1.0
	**10′**	6.7 ± 4.0	20.0 ± 1.1	73.3 ± 3.7
	**20′**	7.0 ± 2.9	13.5 ± 3.8	79.5 ± 5.3
	**40′**	4.8 ± 2.5	12.5 ± 2.8	82.7 ± 5.2
**α-Pol β**	**No**	100 ± 0	0 ± 0	0 ± 0
	**5′**	58.2 ± 5.7	28.6 ± 2.6	13.3 ± 6.2
	**10′**	38.4 ± 3.6	42.0 ± 2.5	19.5 ± 1.1
	**20′**	25.8 ± 3.0	44.7 ± 2.3	29.6 ± 5.2
	**40′**	16.4 ± 0.3	42.4 ± 3.3	41.1 ± 3.0

Supplementary experimental procedures

Creating DNA substrates. The plasmid pGEM3Z.601 (a gift from Jonathon Widom) was used as a template for the PCR-generated substrates [2]. As shown in **Table *S*1**, only the reverse primer is 5' biotinylated. The PCR product was then 5' ^{32}P labeled with ATP [γ-^{32}P] (PerkinElmer) using T4 polynucleotide kinase (Thermo Scientific) on the uracil-containing strand since the biotin at the 5' end of the opposite strand prevents radiolabeling. Gap substrates were created by treating the uracil-containing amplicons with *Escherichia coli* uracil-DNA glycosylase (New England Biolabs) and human AP endonuclease (APE1, New England Biolabs) in 25 mM HEPES pH 7.5, 2mM DTT, 100 µg/mL BSA, 10% glycerol, 5 mM $MgCl_2$, 200 µM EDTA pH8, and 4 mM ATP as previously described [3]. The creation of the nicked THF substrate was similar to the creation of the gap substrates, except the THF-containing aplicon was only treated with APE1. Samples were resuspended in TE buffer (10 mM Tris pH 8.0 and 1 mM EDTA) and subsequently treated with 1 mM spermine pH 7.0 for 1 hour to remove the 5' deoxyribose phosphate moiety [4].

Hydroxyl radical footprinting. Briefly, a 2:1 molar ratio of EDTA disodium salt dihydrate and ammonium iron(II) sulfate was used to generate Fe(II)-EDTA. The •OH radical reaction was initiated when 6 pmol of radiolabeled nucleosome substrate (see "601-147+L50 undamaged" in **Table *S*1**) was incubated in 10 µM of Fe(II)-EDTA, 0.12% H_2O_2, and 1 mM of L(+)-ascorbic acid sodium salt in a total reaction volume of 140 µL. The reaction was allowed to proceed for 10 minutes at room temperature and quenched with glycerol at a final concentration of 6%. Afterwards, histones were removed via PCI (phenol:chloroform:isoamyl alcohol; 25:24:1) extraction and precipitated in ethanol. Samples were resuspended in Hi-Di formamide (Applied Biosystems) and separated on an 8% denaturing polyacrylamide sequencing gel and dried with a gel dryer (Bio-Rad). The gels were exposed to a phosphor screen, scanned via Typhoon FLA 7000 (GE Healthcare Life Sciences), and analyzed by ImageQuant TL (GE Healthcare Life Sciences).

Repair reactions (extended). The SP/LP BER assay was performed by incubating 50 nM of naked or nucleosome gapped DNAs in 50 mM HEPES pH 7.5, 0.5 mM EDTA pH 8.0, 2 mM DTT, 25 mM KCl, 10 mM $MgCl_2$, 10 µM ssDNA 19 mer, 0.1 mM dTTP, 0.1 mM of dideoxy-NTP (ddNTP; the particular ddNTP selected was based on which nucleotide is to be extended after polymerization of the dTTP), 5.2 µg of BTNE, and 1 mM ATP in a 10 µL reaction mix. The samples were treated at 37° for the indicated times and then resuspended in formamide loading buffer (50% formamide and 10 mM EDTA). This was then mixed with 0.1 units of proteinase K (Fermentas) and incubated at 55°C for 15 minutes to digest protein and subsequently incubated at 95°C for 5 minutes to denature DNA. The samples were run at 60 watts on an 8% urea sequencing gel cast in a 21 x 50 cm Sequi-Gen apparatus (Bio-Rad) for 1.25 hours to resolve cleaved and extension products and subsequently dried with a gel dryer (Bio-Rad). The gels were exposed to a phosphor screen, scanned via Typhoon FLA 7000 (GE Healthcare Life Sciences), and analyzed by ImageQuant TL (GE Healthcare Life Sciences). To perform the assay with purified enzymes, BTNE was replaced with purified Pol β and DNA ligase III (gifts from Dr. Rajendra Prasad). The assay to discern BER polymerase extension from the gapped substrates was performed similar to the SP/LP BER assay except ATP was excluded from the reaction.

Neutralizing Pol β assay. Pol β neutralization assays were performed by preincubating equal parts of anti-Pol β (α-Pol β) or pre-immune sera with BTNE on ice for 40 minutes as described previously (see **Supplementary Fig. *S*4a**)[5,6]. Afterwards, 5.2 µg of BTNE was incubated with 50 nM of naked or nucleosome DNAs in 50 mM HEPES pH 7.5, 0.5 mM EDTA pH 8.0, 2 mM DTT, 25 mM KCl, 10 mM $MgCl_2$, 10 µM ssDNA 19 mer, 0.1 mM dTTP, 0.1 mM of ddNTP (the ddNTP is dependent on which nucleotide is to be extended after polymerization of the dTTP) in a 10 uL reaction mix, unless noted in the figure legend. The subsequent steps are as described above.

Immunodepletion. 50 µL of Pan Mouse IgG Dynabeads (Invitrogen) was washed twice with PBS containing 5 mg/mL of BSA. The dynabeads were resuspended in 250 µL of PBS + BSA (5 mg/mL) in the presence or absence of 10 µg of anti-XRCC1 antibody (Abcam, ab1838), and the mixture was rotated overnight at 4°C. The mixture was then washed twice with PBS + BSA. BTNE were then resuspended in immunodepletion buffer (40 mM HEPES, 0.1% Tween-20, 200 mM NaCl, 10% glycerol, and 2x protease inhibitor cocktail (cOmplete protease inhibitor cocktail tablets, Roche)) and dynabeads that were either treated with (ID:XRCC1) or without the anti-XRCC1 antibody (ID:control). The mixture is then rotated overnight at 4°C. Afterwards, the dynabeads were removed from the BTNE and subsequently used in the repair reactions.

Western blot analysis. The ID:XRCC1 and ID:control BTNE were resuspended in SDS loading dye (50 mM Tris-HCl (pH 6.8), 2% SDS, 0.1% bromophenol blue, 10% glycerol, and 100 mM DTT) and run on an 8% SDS-polyacrylamide gel. The proteins were transferred onto a PVDF membrane (Bio-Rad) and probed with either anti-Pol β (Abcam, ab3181) or anti-XRCC1 (Abcam, ab1838) antibodies followed by an anti-mouse secondary antibody (Bio-Rad Lab, 170-6516). The blots

were exposed to Pierce ECL 2 Western Blotting Substrate (Thermo Scientific) followed by detection via Typhoon FLA 7000 (GE Healthcare Life Sciences).

Supplementary references

1 Luger, K., Rechsteiner, T. J. & Richmond, T. J. Preparation of nucleosome core particle from recombinant histones. *Methods Enzymol* **304**, 3-19 (1999).

2 Lowary, P. T. & Widom, J. New DNA sequence rules for high affinity binding to histone octamer and sequence-directed nucleosome positioning. *J Mol Biol* **276**, 19-42, doi:10.1006/jmbi.1997.1494 (1998).

3 Rodriguez, Y. & Smerdon, M. J. The structural location of DNA lesions in nucleosome core particles determines accessibility by base excision repair enzymes. *J Biol Chem* **288**, 13863-13875, doi:10.1074/jbc.M112.441444 (2013).

4 Bailly, V. & Verly, W. G. Possible roles of beta-elimination and delta-elimination reactions in the repair of DNA containing AP (apurinic/apyrimidinic) sites in mammalian cells. *Biochem J* **253**, 553-559 (1988).

5 Singhal, R. K., Prasad, R. & Wilson, S. H. DNA polymerase beta conducts the gap-filling step in uracil-initiated base excision repair in a bovine testis nuclear extract. *J Biol Chem* **270**, 949-957 (1995).

6 Braithwaite, E. K. *et al.* DNA polymerase lambda mediates a back-up base excision repair activity in extracts of mouse embryonic fibroblasts. *J Biol Chem* **280**, 18469-18475, doi:10.1074/jbc.M411864200 (2005).
